# Evaluating Glass Ionomer Cement Longevity in the Primary and Permanent Teeth—An Umbrella Review

**DOI:** 10.3390/jfb15020048

**Published:** 2024-02-19

**Authors:** Alessandro Panetta, Pedro Lopes, Tatiane Fernandes Novaes, Rute Rio, Gustavo Vicentis Oliveira Fernandes, Anna Carolina Volpi Mello-Moura

**Affiliations:** 1 Faculty of Dental Medicine, Universidade Católica Portuguesa, 3504-505 Viseu, Portugal; 2 Center for Interdisciplinary Research in Health, Faculty of Dental Medicine, Universidade Católica Portuguesa, 3504-505 Viseu, Portugal; 3 Faculdade de Odontologia, Universidade Cruzeiro do Sul, São Paulo 04795-902, Brazil; 4 A. T. Still University—Missouri School of Dentistry & Oral Health, St. Louis, MO 63104, USA

**Keywords:** glass ionomer cement, primary teeth, permanent teeth, restorative material, umbrella review

## Abstract

The aim of this umbrella review was to evaluate the longevity of glass ionomer cement (GIC) as a restorative material for primary and permanent teeth. Research in the literature was conducted in three databases (MedLine/PubMed, Web of Science, and Scopus). The inclusion criteria were: (1) to be a systematic review of clinical trials that (2) evaluated the clinical longevity of GICs as a restorative material in primary and/or permanent teeth; the exclusion criteria were: (1) not being a systematic review of clinical trials; (2) not evaluating longevity/clinical performance of GICs as a restorative material; and (3) studies of dental restorative materials in teeth with enamel alterations, root caries, and non-carious cervical lesions. Twenty-four eligible articles were identified, and 13 were included. The follow-up periods ranged from 6 months to 6 years. Different types of GICs were evaluated in the included studies: resin-modified glass ionomer cement (RMGIC), compomers, and low- and high-viscosity glass ionomer cement. Some studies compared amalgam and composite resins to GICs regarding longevity/clinical performance. Analyzing the AMSTAR-2 results, none of the articles had positive criteria in all the evaluated requisites, and none of the articles had an a priori design. The criteria considered for the analysis of the risk of bias of the included studies were evaluated through the ROBIS tool, and the results of this analysis showed that seven studies had a low risk of bias; three studies had positive results in all criteria except for one criterion of unclear risk; and two studies showed a high risk of bias. GRADE tool was used to determine the quality of evidence; for the degree of recommendations, all studies were classified as Class II, meaning there was still conflicting evidence on the clinical performance/longevity of GICs and their recommendations compared to other materials. The level of evidence was classified as Level B, meaning that the data were obtained from less robust meta-analyses and single randomized clinical trials. To the best of our knowledge, this is the first umbrella review approaching GIC in permanent teeth. GICs are a good choice in both dentitions, but primary dentition presents more evidence, especially regarding the atraumatic restorative treatment (ART) technique. Within the limitation of this study, it is still questionable if GIC is a good restorative material in the medium/long term for permanent and primary dentition. Many of the included studies presented a high risk of bias and low quality. The techniques, type of GIC, type of cavity, and operator experience highly influence clinical performance. Thus, clinical decision-making should be based on the dental practitioner’s ability, each case analysis, and the patient’s wishes. More evidence is needed to determine which is the best material for definitive restorations in permanent and primary dentition.

## 1. Introduction

Over time, the evolution of dental materials has occurred because of the change in patient needs, professional perception, and industrial development. In ancient times, an ideal restorative material was seen as stable and passive without interacting with the oral cavity. Over time, the idea began to take hold that active materials, such as the release of fluoride, could have useful effects. They are called “smart materials”, which, by definition, are “materials that have properties which may be altered in a controlled fashion by stimuli, such as stress, temperature, moisture, pH, electric, or magnetic fields” [1]. This led to the development of new materials such as glass ionomer cement (GIC), which has an origin that is associated with this change of approach regarding the qualities demanded in dental material, and the development of new techniques has permitted a better use of it [2]. In fact, GIC can form a specific adhesion with hydroxyapatite and has various clinical applications [3].

The constant need for novelty in dentistry results from this change in professional and patient needs [4]. Dental caries is a highly prevalent disease affecting more than 621-million children worldwide and has been considered an important public health problem. Its treatment is not only technically sensitive but requires a broader approach [5,6], e.g., the use of less invasive techniques, biocompatibility of materials, esthetics, patients’ age and motivation, and lower costs for the patient are all factors that must be taken into consideration for greater clinical success and patient satisfaction [7]. The clinical applications of glass ionomers have been varied: restoration, lining material, sealing, hypersensitivity care, and temporary cavity restoration [8,9]. In fact, it has been claimed that this material acts as an acid buffer with a continuous fluoride release, which may prevent the development of caries lesions in contact with the restoration. In 2018, an international meeting of GIC experts was held, and a reliable GIC should possess some fundamental threshold requirements to achieve this clinical success: (1) compressive strength: the ISO 9917–1:2016 (https://www.gso.org.sa/store/standards/GSO:742697/file/19057/preview (accessed on 11 February 2024)) specification of 100 MPa; (2) microhardness: 70 KHN; (3) acid erosion: the ISO standard, of maximum 0.17 mm; and (4) fluoride release: the highest possible value [10].

Many studies have evaluated the effect of GIC. Frankenberger et al.’s (2009) [11] study enrolled 55 patients who received 108 GICs restorations in permanent molars (21 occlusal and 94 occlusal-proximal restorations). After 24 months, 8% of occlusal restorations and 40% of occlusal-proximal failed. Of these occlusal-proximal failures, 17 were material-associated (nine bulk fractures, four hypersensitivities, three gap formations, one tooth fracture, and one complete loss of the restoration). The authors concluded that occlusal-proximal restorations resulted in extensive wear, fatigue behavior, and insufficient flexural strength [11]. Another study in 2018 included 1.6-million GIC restorations and evaluated their survivability and the factors that might influence it. Regarding the molars enrolled, the survival to re-intervention rate was 81% at 1 year, 52% at 5 years, 36% at 10 years, 28% at 15 years in upper molars, and 81%, 49%, 34%, and 26% at 1, 5, 10, and 15 years in lower molars, respectively [12]. In a study conducted by Ho et al. [13], two conventional GICs were used as restorative materials for 110 small occlusal restorations in molars. There was a 7% early loss of GICs from the occlusal pits and fissures close to the restorations. Only 2.3% of the sealants were totally kept after two years, with 63.2% partially retained. In 2020, a survey was conducted among German dentists to gather information about the type and durability of their permanent molar restorations. Of 1719 molars from 288 dentists, only 0.8% were restored with GIC [14], which is considered a low percentage of the population.

An umbrella review [15] reported that restorative materials with a resin component (resin-modified GICs [RMGIC], composites, and compomers) had similar clinical performance, and they were better when compared to GICs. In addition, the authors showed that only one systematic study included, with a high risk of bias, a comparison between GIC and compomers in Class II restorations with significantly better mean survival time when compomers were applied in primary teeth. Another umbrella study [16] reported using GIC in various forms with the calculated mean failure rate after 2 years ranging between 16% and 21%, and after 3 years, between 16% and 35%, which can be considered significant. For those periods, the lowest failure rates were reported with RMGIC, and the highest failure rates were reported with high-viscosity GICs (HVGIC). Overall, the results found by the authors were considered inconclusive, with most reviews reporting a similar or better performance of GICs compared to conventional restorative materials. Characteristically, GIC had a superior performance for secondary carious lesions.

Due to the weak physical properties of the GIC and some unfavorable results compared to other materials, new additions were inserted in the composition, modifying its structure, such as the addition of metals, fibers, and ceramics, in an attempt to overcome problems and improve the material and clinical performance. The RMGICs were created to overcome the disadvantages of conventional glass ionomers, such as moisture sensitivity and low initial mechanical strength. Clinical applications are the same as conventional GICs, and they are also used in Class I, Class II, and Class III restorations (mostly in primary dentition), as well as Class V restorations as liners and bases, fissure sealants, and bonding agents for orthodontic brackets [17]. Another modification was the metal-reinforced GICs, which added amalgam alloy powder and sintered silver particles to the glass component, improving the diametral tensile strength, hardness, and abrasion resistance [18,19]. Despite these improvements, it also showed failure frequency in terms of the adhesive, cohesive, incidence of brittle fracture in the surface, and less fluoride release [18].

Also, there are hydroxyapatite (HA) and zirconia-reinforced GICs; the HA caused a significant improvement in flexural strength without any significant changes in compressive strength (CS) or diametral tensile strength (DTS) [20], while higher amounts of HA powder (12 and 28%) showed a decline in CS and DTS [20,21]. Otherwise, the addition of nano-HA and nano-fluorapatite (nano-FA) showed improvements in CS, DTS, and biaxial flexural strength (BFS) [22]. The Zirconium (ZrO2) and its subclasses have been used to improve the toughness and strength of brittle HA bioglasses. Studies showed that the ZrO2 addition improved mechanical properties due to the uniformity of particle distribution in the matrix. Also, HA did not dissolve in distilled water. The main weakness was in the interface between ZrO2 and the glass particles, where the propagation of cracks around the glass appeared [23,24]. Recent studies showed that adding nano-zirconia-silica-hydroxyapatite (nanoZrO2-SiO2-HA) to GIC powder improved mechanical and esthetical performance [25,26]. Other types of modifications were yttria-stabilized zirconia-modified GICs [27], fiber-reinforced GICs [28,29,30], zinc-reinforced GICs [31,32], GICs containing YbF3 and BaSO4 [23], niobium pentoxide-modified GICs [33,34], casein phosphopeptide-amorphous calcium phosphate (CPP-ACP)-modified GICs [35,36], silica-reinforced GICs [23,37,38], SrO-reinforced GICs [23,38], fluorinated graphene-modified GICs [39], cellulose nano-crystal (CnCs)-modified GICs [40,41], cellulose nano-crystal (CnCs) and titanium oxide-modified GICs [42], montmorillonite clay-modified GICs [43,44], and forsterite-modified GICs [38].

The choice of materials and treatment options in dentistry should be based on high-quality evidence gathered from high-quality research. A systematic review of systematic reviews, also known as an umbrella review, can be used to filter information by systematically synthesizing data from already published systematic reviews. This type of data synthesis facilitates the identification of eventual research gaps and makes relevant information required by decision-makers and policymakers more accessible. Due to different clinical scenarios, there are several types of glass ionomer and different indications. Knowing how to identify the best GIC for each case is important for the success and longevity of the treatment. Moreover, to the best of our knowledge, this is the first umbrella review approaching exclusively GIC and enrolling deciduous and permanent teeth. Thus, the objective of this study was to evaluate, through an umbrella review, whether primary and permanent teeth can be definitively restored with a GIC, observing the longevity and results found.

## 2. Materials and Methods

This study was registered on the PROSPERO (International Prospective Register of Systematic Reviews) (CRD42022320602). It was reported according to PRIO (Preferred Reporting Items for Overview of Systematic Reviews). Two researchers independently participated in all processes (AP and ACVMM), from article checking, data collection, and risk of bias analysis. A researcher with experience in systematic reviews (TFN) resolved cases of conflict or doubt.

### 2.1. Study Sources

A systematic search of available studies in the literature was conducted in the electronic databases MedLine/PubMed, Web of Science, and Scopus to identify articles. In addition, the reference list of potentially eligible studies was also screened to verify all relevant articles that may not have been identified during the database searches. There was no restriction in the inclusion criteria for the language of publication.

### 2.2. Search Strategy

The search strategies were based on the PICO(S) question, “What is the clinical performance in primary and permanent teeth restored with glass ionomer cement?” The results of the different bases were obtained on 28 April 2022, (without language and date restriction) and crossed to locate and eliminate the duplications. The defined PICO(S) question was: P (patient/problem)—primary and permanent teeth with restorations; I (intervention)—glass-ionomer cement restorations; C (comparison)—comparison between different types of glass ionomers, composite, and amalgam; O (outcome)—restoration longevity; S (study type)—systematic review of clinical trials.

The complete search strategy for MedLine/PubMed was: ((“dental restoration” OR “restoration” OR “atraumatic restorative treatment” OR “permanent teeth” OR “primary teeth”) AND (“glass ionomer cement” OR “glass ionomer”) AND (“success rate” OR “pulp vitality” OR “survival rate”) AND (“systematic review” OR “Syst Rev” OR “overview” OR “review”)).

For the Web of Science database, the following strategy was: TS = ((“dental restoration” OR “restoration” OR “atraumatic restorative treatment” OR “permanent teeth” OR “primary teeth”) AND (“glass-ionomer cement” OR “glass ionomer”) AND (“success rate” OR “pulp vitality” OR “survival rate”) AND (“systematic review” OR “Syst Rev” OR “overview” OR “review”)).

For the Scopus database, the strategy was: TITLE-ABS-KEY = ((“dental restoration” OR “restoration” OR “atraumatic restorative treatment” OR “permanent teeth” OR “primary teeth”) AND (“glass ionomer cement” OR “glass ionomer”) AND (“success rate” OR “pulp vitality” OR “survival rate”) AND (“systematic review” OR “Syst Rev” OR “overview” OR “review”)).

### 2.3. Eligibility Criteria

One reviewer initially evaluated all titles and abstracts of studies found based on the inclusion criteria: (1) to be a systematic review of clinical trials; (2) to evaluate the clinical longevity of GICs in primary and permanent teeth as a restorative material. After the first evaluation, the articles that met the inclusion criteria were reviewed in their entirety, with those that presented at least one of the following exclusion criteria being excluded: studies of dental restorative materials in teeth with the presence of root caries and non-carious cervical lesions. The full papers of the included studies were read to ensure that they were about the restoration of primary and permanent teeth with GICs and were not critical/narrative reviews, letters to the editor, or guidelines.

### 2.4. Data Collection

The same reviewers (ACVMM and AP) collected the data independently in tables structured in Excel spreadsheets (Microsoft Office, Microsoft Corporation, California, USA). The information extracted was: title, year, authors, PICO, protocol record (yes or no), number of included studies, statistical analyses (meta-analysis), databases used, search strategy, search date, number of reviewers, inclusion and exclusion criteria, language, type of restorative materials, restorative technique, follow-up period, objective of the study, quality analysis and risk of bias (yes or no and which tools were used), main results, and conclusions.

### 2.5. Quality Assessment and Risk of Bias

Two reviewers (ACVMM and AP) independently performed the quality and risk of bias analyses. The included studies’ methodological quality was measured using the AMSTAR-2 tool [45], while the ROBIS tool [46] was used to assess the risk of bias.

The GRADE tool was implemented, and the evidence and class of recommendations were assessed. The level of evidence was divided into (A) data derived from multiple randomized clinical trials or meta-analyses; (B) data derived from a single randomized clinical trial or large non-randomized studies; (C) consensus experts’ opinion and/or small studies, retrospective studies, and registries. The classes of recommendations followed the definition: Class 1—evidence and/or general agreement that a particular treatment or procedure is beneficial, useful, and effective (it is recommended/is indicated); Class II—conflicting evidence and/or a divergence of opinion about the usefulness/efficacy of the particular treatment or procedure; Class IIa—weight of evidence/opinion is in favor of usefulness/efficacy (it should be considered); Class IIb—usefulness/efficacy is less well established by evidence/opinion (it may be considered); and Class III—evidence or general agreement that the particular treatment or procedure is not useful/effective, and in some cases may be harmful (it is not recommended) [47].

## 3. Results

### 3.1. Study Selection

**Figure 1:**
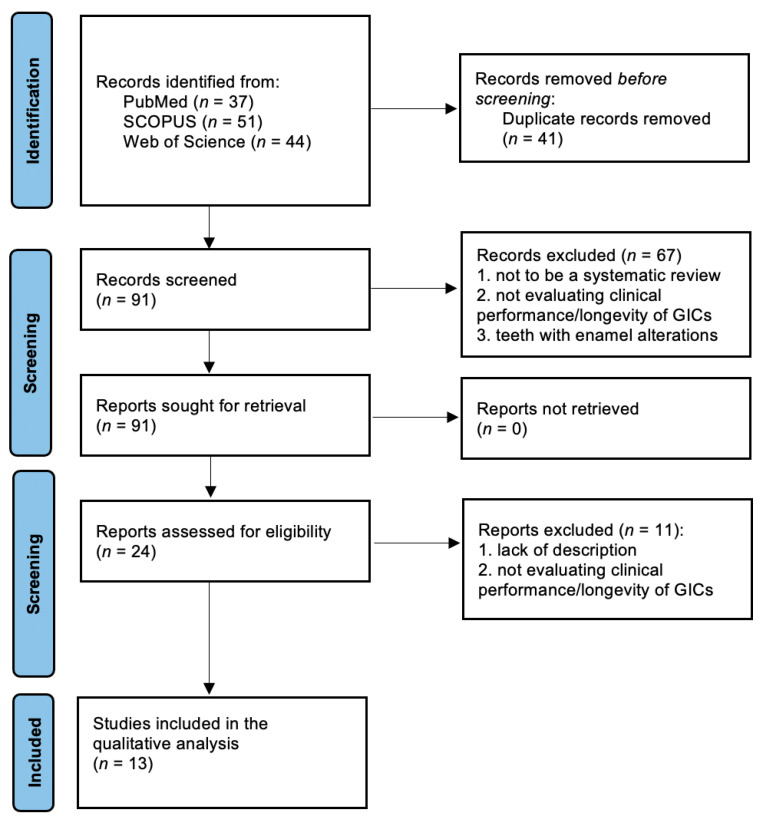
Overview of article-selection procedure according to Preferred Reporting Items for Systematic Reviews and Meta-Analyses (PRISMA).

A systematic literature search identified 132 references to potentially relevant studies (37 publications from MedLine/PubMed, 51 from Scopus, and 44 from Web of Science). Duplicates were excluded (*n* = 41). Based on the information provided in the title and abstract, 67 articles were considered ineligible. The main reasons for non-inclusion were: (1) not a systematic review, (2) not evaluating clinical performance/longevity of GICs, and (3) teeth with enamel alterations were observed. Twenty-four articles were fully analyzed to collect more detailed information. In this step, 11 studies were excluded for the following reasons: (1) not being a systematic review of clinical trials and (2) not evaluating clinical performance/longevity of GICs. Finally, 13 studies [48,49,50,51,52,53,54,55,56,57,58,59,60] were included in the following review. The study selection process is shown in Figure 1.

### 3.2. Characteristics of Included Studies and Quality Assessment

The characteristics of the included studies are shown in Table 1. Only six studies presented the PICO question, and seven mentioned protocol registration. Two studies did not report a search strategy. All the studies presented inclusion and exclusion criteria. The follow-up periods ranged from 6 months to 6 years. Regarding quality assessment and risk of bias, only six studies presented both, whereas two did not present anything. Two studies presented quality analysis but not risk of bias, and the other two presented the opposite. Four studies included results in other languages besides English. The included studies evaluated the clinical performance of GICs used as a definitive restorative material in both primary and permanent dentitions. Different types of GICs were evaluated in the included studies: resin-modified glass-ionomer cement (RMGIC), compomers, and low- and high-viscosity glass-ionomer cement. Some studies compared amalgam and composite resins to GICs regarding longevity/clinical performance.

Analyzing the AMSTAR-2 results (Table 2), none of the articles had positive criteria in all the evaluated requisites, and none of the articles had an a priori design. Most of the studies [48,49,50,51,52,53,54,55,58,59,60] defined a search strategy (positive criteria) and excluded grey literature [48,52,54,55,56,57,58,59]. Inclusion and exclusion criteria were described in all the studies, receiving a positive value. Four studies [53,54,57,60] did not use tools for quality analysis, being negatively evaluated. Two studies [57,60] did not use any risk-of-bias tool. Two studies [58,60] have some conflict of interest. However, they are more related to teaching the use of GIC in different strategies.

Specific points were analyzed for the quality of the studies. Some of them reduced the quality level; for example, none of them provided an “a priori” design for the study performed, and two studies, Amorim et al. [58] and Mickenautsch et al. [60], declared a conflict of interest with the study developed. Also, three studies did not control the publication bias [57,59,60], and four of them failed to document the scientific quality of the included articles [53,54,57,60].

### 3.3. Risk of Bias

The criteria considered for the analysis of the risk of bias of the included studies were evaluated through the ROBIS tool, and the results of this analysis are described in Table 3. Two studies [49,51] presented a very low risk of bias. Meanwhile, three studies [48,50,58] showed positive results in all criteria except for one criterion of unclear risk. One study [57] showed a very high risk of bias, with negative results in all criteria. Five studies [52,54,56,59,60] showed three positive results out of five and can be considered a low risk of bias. Ruengrungsom et al. [53] showed high risk of bias.

### 3.4. Quality Assessment Analysis

**Figure 2:**
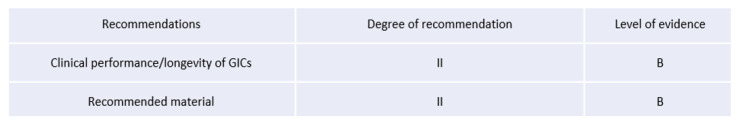
Results of quality-of-evidence analysis.

The GRADE tool was used to determine the quality of the included studies’ evidence. The degree of recommendations was classified as Class II, meaning there was still conflicting evidence on the clinical performance/longevity of GICs and their recommendations compared to other materials. The level of evidence is classified as Level B, meaning that the data were obtained from less robust meta-analyses and single randomized clinical trials. Results are shown in Figure 2.

## 4. Discussion

This umbrella review aimed to gather the maximum level of scientific evidence to determine if GICs are a good option to restore permanent and primary teeth in a definitive way. To the best of our knowledge, this is the first umbrella review approaching exclusively GIC in permanent teeth. Therefore, it is crucial to understand if glass-ionomer cements have enough scientific evidence to make them a safe choice for definitive restorations of primary and permanent teeth and compare their clinical performance to other dental materials. Similar studies were published but presented a larger approach for restorative materials or focused on compomers [15,16], always studying the application on primary teeth. Andas et al. [15] concluded that compomers were similar to other dental-filling materials for the placement of direct restorations in primary teeth, whereas Amend et al. [16] reported that all materials studied had acceptable mean failure rates and could be recommended for the restoration of carious primary teeth. Thus, our umbrella review is aimed to study only the GIC application on both dentitions (primary and permanent).

Observing the degree of recommendations of all studies included in this umbrella review (*n* = 13), they were classified as Class II, which means there was still conflicting evidence on the clinical performance/longevity of GICs and their recommendations compared to other materials. In addition, the level of evidence of the included revisions was classified as Level B, which means that the data were obtained from less robust meta-analyses and single randomized clinical trials. The studies classified as “high quality” showed robust methodology, an organized selection of the studies, and an elaboration of the findings. On the other hand, low-quality studies were lacking in methodologies, such as quality analysis, risk of bias, and study eligibility criteria, or they were unclear about the findings, making them unreliable to advise any material.

The number of dental restorative materials introduced into the market in the last few years has grown rapidly [61]. Then, choosing the restoration material is important and depends on the clinical case. Conventional restorative materials (amalgam and resin composites) have a limited application in primary teeth but are highly applied in permanent teeth. Despite those materials having an acceptable annual failure rate, their use in everyday practice is reduced in primary teeth. This fact can be attributed to low longevity, which is directly associated with patient-related factors. GIC has been recommended due to results that demonstrated similarity regarding the annual failure rate of GIC and conventional restoration with composite or amalgam [53], and because it is a less technique-sensitive material, presenting high success rates and biocompatibility with an easier and faster/lesser time-consuming application as compared to resin composites. These facts improve the procedure’s acceptability by the patients and have a positive effect on behavioral shaping and overall management of even uncooperative patients [62]. Moreover, its adhesion to the tooth is comparable to the retention reached by composite, providing similar longevity rates [63].

In addition, composite restorations are highly sensitive to moisture control, which may jeopardize their performance [64]. This sensitivity can increase the prevalence of restoration loss. Similarly, with amalgam, even though it possesses a high durability with a survival range up to 7 years, there is concern that it contains specific classes of cavities in primary teeth due to its toxicity and the lack of esthetics [65]. In our umbrella review, the present results emphasized that more high-quality studies are needed to evaluate the GICs’ performance as a restorative material.

In permanent dentition, conventional HVGIC restorations showed high survival rates (2–6 years) regarding surface texture, marginal discoloration and adaptation, and anatomic form [53]. Another study reported high survival rates regarding ART/HVGIC approach in posterior permanent teeth over the first 5 years in single-surface restorations, while it was not possible to conclude for multi-surface restorations [58]. ART techniques showed a wide range of survival rates (29.6–100%) between 4-month and 6-year follow-ups regarding single occlusal restorations and between 6-month and 2-year follow-ups in multi-surface restorations (30.6–100%) [57].

In primary dentition, the atraumatic restorative treatment (ART) technique can be effective because it helps reduce the anxiety of the patients [49,50,58]. Despite wide application in primary dentition, two studies found that ART’s annual failure rates (AFRs) were higher in primary dentition than in permanent detention in single- and multi-surface restorations [49,53]. The main reasons for failure were marginal defects, loss of the restoration, excessive wear, and retention loss in both dentitions [50,53,54]. There is conflicting evidence regarding conventional GIC restoration techniques compared to ART-GIC restoration techniques since different studies showed better results with conventional techniques and no significant differences, respectively [53,58,59]. ART is a better choice in single-surface occlusal restorations [59]. ART techniques generally show high survival rates in both dentitions after 3–6 years.

High-viscosity glass-ionomer cements with a resin coating (HVGIC/RC) seem to have similar results compared to conventional GICs and composite resin up to 5 years in Class I and II restorations in terms of fracture toughness, retention rates, and abrasion resistance [56]. Only one study reported inferior longevity of HVGICs compared to composite resins [54]. Glass-ionomer cements have evolved over the years, and several modifications have been experimented with, but there is not enough scientific evidence on their effectiveness to be used in clinical situations. One modification actively used in patients is resin-modified glass-ionomer cement (RMGIC), which is used in both primary and permanent dentitions [50,53].

RMGICs seem to be a very good option in restoring Class I and II cavities and performed better than high-viscosity glass-ionomer cement (HVGIC) and composite resins in some studies in terms of survival rates, fluoride release, and biologic considerations [48,51,53]. Other studies could not find significant differences between RMGICs compared to conventional GICs, composite resins, compomers, and amalgam [50,51,52,53,59], or there is conflicting evidence on which performs better between RMGICs and composite resins. Manisha et al. [61] demonstrated that RMGIC and high-viscosity GIC had less favorable compressive strength and microleakage performance when compared with zirconia-reinforced glass-ionomer cement. Moreover, compomers and GICs had greater compressive strength and reduced microleakage values than zirconia-reinforced GICs.

Silver-reinforced glass-ionomer cement (SRGIC) had lower survival rates and higher recurrences of secondary caries [48]. Compomers seem statistically better than conventional GICs regarding median survival time (MST), surface texture, marginal discoloration, tooth decay, and higher fatigue and fracture resistance, while RMGICs showed no significant differences [51]. In other studies, the compomer performed better than conventional GIC and RMGIC in terms of survival rates, marginal adaptation, surface roughness, and form [53,55].

There is conflicting evidence on the best performance between conventional GICs and composite resins. In all studies, Class II restorations showed higher failure rates than Class I restorations in both dentitions, irrespective of materials or techniques. Then, the type of cavity and the operator’s experience highly influenced the survival rates. Most of the evidence is aimed at primary dentition.

The choice of the “best material” depends on the situation since every clinical case is unique, with many different variables to be considered; for example, setting, permanent or primary dentition, operator experience, type, and location of the cavity. The material of choice should be evaluated as a result of these factors.

### 4.1. Limitations of this Study

This was an umbrella review that critically appraised the accessible evidence and presented a comprehensive overview of the GIC longevity in the primary and permanent teeth. Thirteen studies were enrolled in which discrepancies were observed. Firstly, there was heterogeneity in the study design included in the systematic reviews. Also, the type/composition of GIC varied among them. Moreover, they included between three and 67 articles (six included up to 11 studies; three included between 15 and 24 studies; and four included between 34 and 67 studies); this fact shows a discrepancy among them for the criteria used. Furthermore, five of them included up to two databases, which can be considered limited compared to the other included studies; two articles had an incomplete presentation of the search strategy; six did not report the remotion of the duplicated articles; and one did not mention the number of reviewers for the study selection. Three studies did not have a risk of bias developed; three did not have a quality assessment; and five were considered as an extremely short-term evaluation (at least 4 and 6 months). Also, various outcome measures precluded the quantitative synthesis of results. Ultimately, the unclear or high risk of bias among most included systematic reviews ruled out meta-analyses.

The data become even more heterogeneous by using different evaluation criteria with various cut-off points to evaluate the longevity of restorations. The quality of evidence is further restricted with the finding that several systematic reviews performed meta-analyses based on primary studies with an overall unclear or high risk of bias. Therefore, the potential risk of bias in the measurement of the outcome has been considered when interpreting the outcome of these studies.

### 4.2. Recommendations for Future Studies

There is a need for further well-designed clinical studies, specifically randomized clinical trials, to overcome the limitations of studies on the restorative treatment of carious lesions and to increase the internal validity. More long-term studies are also needed to evaluate if GICs are a good choice for extended periods (>6 years). The trials should be conducted with adequate random-sequence generation and allocation-sequence concealment to avoid bias arising from the randomization process.

Future sample-size calculations based on power analyses should consider the high dropout rate observed among primary studies to obtain meaningful results after more extended follow-up periods. The caries risk of participants should be reported to correlate the higher caries risk with increased susceptibility to restoration failure. As far as the teeth are concerned, the included teeth, the cavity class, and the caries depth and extension should be mentioned. The experience of the operators performing the restorative treatment should be mentioned because it may directly influence the clinical performance of restorations. Reporting treatment-related factors, such as the isolation technique and detailed descriptions of restorative materials and techniques, facilitates the interpretation of results, which is desirable for future studies. In addition to the classical outcome measures, patient-related and reported factors, such as discomfort, pain, and the impact on the oral health-related quality of life, should be further documented.

## 5. Conclusions

Within the limitations of this study, it is still questionable if GIC is a good restorative material in the medium/long term for permanent and primary dentition. Many of the studies included presented a high risk of bias and low quality. The techniques, type of GIC, type of cavity, and operator experience highly influence clinical performance. Thus, clinical decision-making should be based on the dental practitioner’s ability, each case analysis, and the patient’s wishes. Then, more evidence is needed to determine the best material for definitive restorations in permanent and primary dentition. Due to the lack of studies comparing the performance of different dental restorative materials, it is not possible to conclude whether GICs perform better or worse than other materials.

## Figures and Tables

**Table 1:** Characteristics of the included studies.

Author (year)	Title	Objective of the Study		Pico	Protocol Registration	Articles Included	Meta-Analysis	Databases	Search Strategy	Duplicate Elimination	Included Languages	Number of Reviewers	Inclusion Criteria	Exclusion Criteria
Santamaría et al., 2020 [48]	How to Intervene in the Caries Process: Dentin Caries in Primary Teeth	To evaluate the treatment and material used in primary dentition to effectively treat dentin carious lesions		Yes	No	18	No	MEDLINE/PubMed	Yes	Not specified	English, Spanish	6	Yes	Yes
Garbim et al., 2021 [49]	Atraumatic Restorative Treatment Restorations Performed in Different Settings: Systematic Review and Meta-analysis	To evaluate the atraumatic restorative treatment (ART) longevity in primary and permanent dentition.		Yes	Yes	34	Yes	PubMed/MEDLINE, Scopus, Web of Science, Open Grey	Yes	Yes	English, no restrictions	3	Yes	Yes
Maia et al., 2021 [50]	Survival of Atraumatic Restorative Treatment Restorations in the Elderly Patients: A Systematic Review	To evaluate the atraumatic restorative treatment (ART) longevity in elderly patients.		Yes	Yes	7	No	PubMed/MEDLINE, Scopus, LILACS, SciELO, Embase, Web of Science, OpenGrey, OpenThesis	Yes	Yes	English, Portuguese, no restrictions	2	Yes	Yes
Santos et al., 2016 [51]	Survival of Adhesive Restorations for Primary Molars: A Systematic Review and Meta-analysis of Clinical Trials	To evaluate the longevity and clinical performance of different adhesive restorative materials in primary molars.		Yes	Yes	11	Yes	Cochrane Oral Health Group’s Trials Register, PubMed/MEDLINE, Web of Science, Cochrane Library, LILACS, Clinical Trials—U.S. National Institute of Health, National Institute for Health and Clinical Excellence	Yes	Yes	English, no restrictions	6	Yes	Yes
Yengopal et al., 2009 [52]	Dental Fillings for the Treatment of Caries in the Primary Dentition	To compare the outcomes of different dental materials used in restorations of carious lesions in primary dentition.		No	Yes	3	No	The Cochrane Oral Health Group’s Trials Register, PubMed Central, PubMed/Medline, EMBASE, SIGLE	Yes	Yes	English, no restrictions	2	Yes	Yes
Ruengrungsom et al., 2018 [53]	Comparison of ART and Conventional Techniques on Clinical Performance of Glass-ionomer Cement Restorations in Load-bearing Areas of Permanent and Primary Dentitions: A Systematic Review	To observe the clinical performance of glass-ionomer cement in Classes I and II restorations using the ART techniques.		No	No	67	No	PubMed	Yes	Not specified	English	Not specified	Yes	Yes
Heintze et al., 2022 [54]	Clinical Efficacy of Resin-based Direct Posterior Restorations and Glass-ionomer Restorations—An Updated Meta-analysis of Clinical Outcome Parameters	To evaluate the longevity of Class I and II restorations performed with resin-based materials and glass ionomers.		Yes	No	62	Yes	PubMed, SCOPUS	Yes	Not specified	English	2	Yes	Yes
Tedesco et al., 2018 [55]	Scientific Evidence for the Management of Dentin Caries Lesions in Pediatric Dentistry: A Systematic Review and Network Meta-analysis	To observe the success rate and effectiveness of dentin carious lesion treatments in primary dentition.		Yes	Yes	15	Yes	MEDLINE/PubMed, Web of Science, Scopus	Yes	Yes	English, no restrictions	3	Yes	Yes
Kielbassa et al., 2016 [56]	Systematic Review on Highly Viscous Glass-ionomer Cement/Resin Coating Restorations (Part I): Do They Merge Minamata Convention and Minimum-intervention Dentistry?	To evaluate the clinical performance of high-viscosity glass-ionomer cement/resin coating (hvGIC/RC) in class I and II.		Yes	No	7	No	PubMed, Cochrane Library, EBSCO, EMBASE, SCOPUS	No	Yes	English	5	Yes	Yes
Studart et al., 2012 [57]	Atraumatic Restorative Treatment in Permanent Molars: A Systematic Review	To evaluate clinical performance of ART technique in permanent molars.		No	No	24	No	PubMed/MEDLINE, LILACS	No	Not specified	English, Portuguese, Spanish	3	Yes	Yes
Amorim, 2018 [58]	Survival Percentages of Atraumatic Restorative Treatment (ART) Restorations and Sealants in Posterior Teeth: An Updated Systematic Review and Meta-analysis	To assess ART restorations and sealants survival rates and carious preventive effects in permanent and primary posterior teeth.		No	Yes	43	Yes	PubMed, EMBASE, LILACS, BBO, CNKI, VIP	Yes	Not specified	English, Dutch, Spanish, Portuguese, Chinese	6	Yes	Yes
Raggio et al., 2012 [59]	Is Atraumatic Restorative Treatment an Option For Restoring Occlusal-proximal Caries Lesions in Primary Teeth? A Systematic Review and Meta-analysis	To evaluate if ART is a viable option for occlusal-proximal restorations in the primary dentition.		No	No	3	Yes	PubMed	Yes	Not specified	English	3	Yes	Yes
Mickenautsch et al., 2015 [60]	Failure Rate of Direct High-Viscosity Glass-ionomer Versus Hybrid Resin Composite Restorations in Posterior Permanent Teeth—a Aystematic Review	To evaluate the success rates/longevity of high-viscosity glass-ionomer cement compared to hybrid composite resins in single/multi-surface restorations in permanent posterior teeth.		Yes	Yes	6	No	PubMed/Medline, CENTRAL (Cochrane Library), Directory of Open Access Journals (DOAJ), Biomed Central, IndMed, Sabinet, OpenSIGLE, GoogleScholar	Yes	Yes	English	2	Yes	Yes
**Author (year)**	**Material Type**	**Follow-Up**	**Quality Analysis**	**Risk of Bias**	**Tools/Data Analysis**			**Outcome**				**Conclusions**		
Santamaría et al., 2020 [48]	Preformed metal crowns, amalgam, composite resin, glass ionomer cement, and compomer	At least 12 months after intervention	Yes	Yes	PRISMA statement, Cochrane risk of bias tool, Mendeley software, ORCA/EFCD consensus workshop			The studies included compared different dentin carious lesions techniques and approaches, such as selective carious tissue removal, no carious tissue removal, atraumatic restorative treatment (ART), ultraconservative treatment (UCT), and no treatment of carious lesions. Also, results compared the performance of different restorative dental materials.				Due to insufficient evidence and limited quality, a conclusion could not be drawn. But in general, there is not an ideal single treatment that must be considered when managing dentin carious lesions in primary teeth.		
Garbim et al., 2021 [49]	High-viscosity glass ionomer	12 or 36 months	Yes	Yes	PRISMA guidelines 2020, RoB 2.0 tool—Cochrane Handbook for Systematic Reviews of Interventions, ROBINS-I tools, Begg’s Test, Rstudio, ART evaluation criteria, United States Public Health Service (USPHS), GemertSchriks criteria			Different brands of glass-ionomer cements were compared regarding ART longevity in primary and permanent dentition, as well as in occlusal and multi-surface restorations.				ART restorations seem to have long-term survivability, making them reliable treatments in both primary and permanent dentition.		
Maia et al., 2021 [50]	Glass ionomer	6 months–5 years	Yes	Yes	Joanna Briggs Institute’s Critical Appraisal Tool, PRISMA guidelines			Conventional and resin-modified glass ionomer cements (RMGIC) were compared in terms of ART survivability, evaluating wear, marginal defects, and need for replacement.				Even though it would be ideal to conduct more studies with longer follow-ups, ART showed positive results in terms of longevity in elderly patients.		
Santos et al., 2016 [51]	Composite resin (CR), conventional glass-ionomer cement (GIC), resin-modified glass ionomer (RMGIC), silver-reinforced glass-ionomer cement, and compomer	18, 24, 36 months	Yes	Yes	PRISMA guidelines, Reference Manager 12.03.0, modified version of the Jadad scale (0–6 points), comprehensive Meta-Analysis Program (Biostat, Englewood, N.J., USA), United States Public Health Service (U.S.PHS)			Results compared how five different restorative materials performed in Classes I and II in 3–10-year-old patients in different settings, with different types of isolations.				More randomized controlled trials are needed, but from this study, all materials performed well when used for restorations in primary molars, except the silver-reinforced glass-ionomer cement, which had the worst survival rate. Glass-ionomer cements did not show lower survival rates compared to resin-based materials.		
Yengopal et al., 2009 [52]	Resin-modified glass ionomer, amalgam, compomer	Minimum 6 months	Yes	Yes	Cochrane Collaboration statistical guidelines, Cochrane Highly Sensitive Search Strategy (CHSSS), Cochrane Handbook for Systematic Reviews of Interventions 5.0.1, Chi^2^ test for heterogeneity, Ryge criteria			Resin-modified glass-ionomer cement compared to amalgam in Class II restorations. The clinical success was evaluated by considering surface texture, marginal integrity, axial contour, wear, restoration placement, secondary caries, restoration fracture, and staining.				Due to the absence of scientific evidence, it is not possible to recommend a specific material. The three trials did not show any significant differences regarding the outcomes.		
Ruengrungsom et al., 2018 [53]	Glass-ionomer cements	>1 year	No	Yes	ROBINS-I, PRISMA flow diagram			Comparison of single-/multi-surface restorations in load-bearing areas in permanent and primary teeth performed with conventional GIC technique versus ART technique and reasons for failure.				The conventional GIC technique showed better results compared to the ART technique, and it is preferred in primary dentition (lower annual failure rates). In general, multi-surface restorations showed greater failure rates compared to single-surface restorations. The main causes of failures were fracture and dislodgment. The RMGIC conventional technique showed promising results in restoring proximal cavities.		
Heintze et al., 2022 [54]	Resin composite, compomer, or GIC restorations (high-viscous glass ionomer and resin-modified glass ionomer)	>2 years	No	Yes	Ryge criteria, Cochrane Collaboration’s tool			In the analysis, materials were divided into microhybrid, nanohybrid, and hybrid. Regarding composite filler and bulk fill materials, GICs and compomers were treated as separate categories. The conditioning and adhesive systems were: etch and rinse two and three steps, self-etch two and three steps, enamel etch and enamel bonding, and no etching/adhesive systems (polyacrid acid and GICs). Curing time ranged from 10–60 s. Follow-up periods ranged from 2–5 years.				Resin showed better results in terms of longevity compared to compomers and GICs due to fracture and excessive wear.		
Tedesco et al., 2018 [55]	Low- and high-viscosity glass-ionomer cement, resin-modified glass-ionomer cement (RMGIC), resin composite, amalgam	>1 year	Yes	Yes	Cochrane Handbook for Systematic Reviews of Interventions, ROBINS-I, PRISMA-NMA extension, GRADE tool, I^2^ test, R package “stats” version 2.15.3			Comparison between conventional restorative treatment (CRT), atraumatic restorative treatment (ART), non-restorative caries treatment (NRCT), and ultraconservative caries treatment (UCT) in occlusal/occlusal-proximal surfaces in primary dentition.				The success of the treatment depends on the depth of progression of the caries and the surfaces involved. Without information about the depth of progression of the carious lesions, the CRT technique with compomer showed the best results followed by the ART technique. The Hall technique (stainless-steel crowns) performed best in occlusal-proximal surfaces. Application of 38% silver diamine fluoride (SDF) twice per year showed a great increase in caries reduction. In the end, it is not possible to recommend the best treatment option due to few studies with a high risk of bias.		
Kielbassa et al., 2016 [56]	High-viscosity glass-ionomer cement/resin coating	6 months–6 years	Yes	Yes	Oxford quality-scoring system, PRISMA guidelines, US Public Health Service (USPHS) criteria			Resilience, wear, durability, abrasion resistance, chipping, color match, and marginal adaptation were considered when assessing the clinical performance of hvGIC/RC in Class I and II restorations.				Short–medium-term hvGIC/RC systems showed promising results in terms of abrasion resistance, retention rates, and clinical fracture toughness with high survival rates in both Class I and II restorations, although less longevity was shown in Class II compared to Class I.		
Studart et al., 2012 [57]	Resin-modified glass-ionomer cements, low- and high-viscosity glass-ionomer cements, amalgam	>4 months	No	No	Not specified			Different ART restorative materials and methods in single- and multi-surface cavities in permanent molars were compared.				ART showed high survival rates in single (3 years) and multi-surface (2 years) restorations. Longevity in multi-surface restorations was lower compared to single surface. However more studies are required to draw conclusions.		
Amorim, 2018 [58]	Low- and high-viscosity glass-ionomer cements	>1 year	Yes	Yes	PRISMA statement, I^2^ values, Cochrane Research Group			The survival rates of single-surface ART restorations in permanent posterior teeth were 87.1% over 3 years and 77% for multi-surface over 5 years. In primary dentition for single-surface restorations, the survival rate was 94.3%, and for multi-surface restorations, it was 65.4% over 2 years. The mean annual dentine carious lesion failure rates were 0.9% over 3 years and 1.9% over 5 years.				Both dentine carious lesion preventive effects and survival rates over 2–5 years of ART restorations in primary and permanent dentitions were relatively high, with the exception of restorations in primary posterior teeth over 2-year period, which showed lower survival rates. In general, multi-surface restorations present lower survival rates compared to a single surface.		
Raggio et al., 2012 [59]	High-viscosity glass-ionomer cement	>6 months	Yes	No	Comprehensive Meta Analysis 2.2.064, US Public Health Service (USPHS) criteria			ART with high-viscosity glass-ionomer cements and conventional restorative techniques with amalgam or composite resin were compared, resulting in 48.7–88.9% survival rates in ART restorations and 42.9–100% in conventional restorations.				ART and conventional restoration techniques showed similar success rates and are a viable option for occlusal-proximal restorations in primary molars. More studies on factors such as pulp damage and caries lesion progression are needed.		
Mickenautsch et al., 2015 [60]	High-viscosity glass-ionomers, hybrid resin composites	>1 year	No	No	Australian New Zealand Clinical Trials Registry, Clinical Trials US, EU Clinical Trials Register, metaRegister of Controlled Trials (mRCT), South African National Clinical Trials Register, WHO Clinical Trials, IADR abstracts, International Poster Journal of Dentistry and Oral Medicine, Rev Man 4.2, Berger VW. Selection bias and covariate imbalances in randomised clinical trials. Chichester, UK: John Wiley & Sons, Ltd. 2005, ITC software, I^2^ test, Cochrane’s Q-test			The statistical comparison between hvGIC and HRC in Class I and II restorations in permanent posterior teeth after 24–60 months showed no differences.				No final judgements can be made due to poor scientific evidence on this comparison. More studies are needed to draw conclusions.		

**Table 2:** Criteria adopted for analyzing the methodological quality of the studies and their respective responses (AMSTAR-2 tool).

Criteria	Was an ‘a Priori’ Design Provided?	Was There Duplicate Study Selection and Data Extraction?	Was a Comprehensive Literature Search Performed?	Was the Status of Publication (i.e., Grey Literature) Used as an Inclusion Criteria?	Was a List Od Studies (Included and Excluded) Provided?	Were the Characteristics of the Included Studies Provided?	Was the Scientific Quality of the Included Studies Assessed and Documented?	Was the Scientific Quality of the Included Studies Used Approprietely in Formulating Conclusions?	Were the Methods Used to Combine the Findings of Studies Appropriate?	Was the Likelihood of Publication Bias Assessed?	Was the Conflict of Interest Stated?
**Author/Year**											
1. Santamaría et al., 2020 [48]	No	No	Yes	No	Yes	Yes	Yes	Yes	Yes	Yes	No
2. Garbim et al., 2021 [49]	No	Yes	Yes	Yes	Yes	Yes	Yes	Yes	Yes	Yes	No
3. Maia et al., 2021 [50]	No	Yes	Yes	Yes	Yes	Yes	Yes	Yes	Yes	Yes	No
4. Santos et al., 2016 [51]	No	Yes	Yes	No	Yes	Yes	Yes	Yes	Yes	Yes	No
5. Yengopal et al., 2009 [52]	No	Yes	Yes	No	Yes	Yes	Yes	Yes	Yes	Yes	No
6. Ruengrungsom et al., 2018 [53]	No	No	Yes	No	Yes	Yes	No	Yes	No	Yes	No
7. Heintze et al., 2022 [54]	No	No	Yes	No	Yes	Yes	No	Yes	No	Yes	No
8. Tedesco et al., 2018 [55]	No	Yes	Yes	No	Yes	Yes	Yes	Yes	Yes	Yes	No
9. Kielbassa et al., 2016 [56]	No	Yes	No	No	Yes	Yes	Yes	Yes	Yes	Yes	No
10. Studart et al., 2012 [57]	No	No	No	No	Yes	Yes	No	Yes	No	No	No
11. Amorim et al., 2018 [58]	No	No	Yes	No	Yes	Yes	Yes	Yes	Yes	Yes	Yes
12. Raggio et al., 2012 [59]	No	No	Yes	No	Yes	Yes	Yes	Yes	No	No	No
13. Mickenautsch et al., 2015 [60]	No	Yes	Yes	Yes	Yes	Yes	No	Yes	No	No	No

**Table 3:** Results of risk-of-bias assessment using the ROBIS tool. Green = low risk of bias; Red = high risk of bias; ? (blue) = moderate risk of bias.

	Phase 2				Phase 3
Criteria	Study Eligibility	Identification and Selection of Studies	Data Collection and Study Appraisal	Synthesis and Findings	Risk of Bias in the Review
**Author/Year**					
1. Santamaría et al., 2020 [48]					?
2. Garbim et al., 2021 [49]					
3. Maia et al., 2021 [50]					?
4. Santos et al., 2016 [51]					
5. Yengopal et al., 2009 [52]					?
6. Ruengrungsom et al., 2018 [53]					
7. Heintze et al., 2022 [54]	?				
8. Tedesco et al., 2018 [55]					
9. Kielbassa et al., 2016 [56]					
10. Studart et al., 2012 [57]					
11. Amorim et al., 2018 [58]	?				
12. Raggio et al., 2012 [59]					
13. Mickenautsch et al., 2015 [60]					

## Data Availability

All data were inserted in the article.
